# Stress-induced microglial activation occurs through β-adrenergic receptor: noradrenaline as a key neurotransmitter in microglial activation

**DOI:** 10.1186/s12974-019-1632-z

**Published:** 2019-12-17

**Authors:** Shuei Sugama, Takato Takenouchi, Makoto Hashimoto, Hisayuki Ohata, Yasuhiro Takenaka, Yoshihiko Kakinuma

**Affiliations:** 1 Department of Physiology, Nippon Medical School, 1-1-5 Sendagi Bunkyo-ku, Tokyo, 113-8602 Japan; 2 Institute of Agrobiological Sciences, National Agriculture and Food Research Organization, 1-2 Ohwashi, Tsukuba, Ibaraki, 305-8634 Japan; 3 Division of Sensory and Motor Systems, Tokyo Metropolitan Institute of Medical Science, 2-1-6 Kamikitazawa, Setagaya-ku, Tokyo, 156-0057 Japan

**Keywords:** Microglia, Restraint stress, Brain, CNS, Neuroimmunomodulation

## Abstract

**Background:**

The involvement of microglia in neuroinflammatory responses has been extensively demonstrated. Recent animal studies have shown that exposure to either acute or chronic stress induces robust microglial activation in the brain. In the present study, we investigated the underlying mechanism of brain microglial activation by acute stress.

**Methods:**

We first looked at the spatial distribution of the noradrenaline (NA)-synthesizing enzyme, DBH (dopamine β-hydroxylase), in comparison with NA receptors—β1, β2, and β3 adrenergic receptors (β1-AR, β2-AR, and β3-AR)—after which we examined the effects of the β-blocker propranolol and α-blockers prazosin and yohimbine on stress-induced microglial activation. Finally, we compared stress-induced microglial activation between wild-type (WT) mice and double-knockout (DKO) mice lacking β1-AR and β2-AR.

**Results:**

The results demonstrated that (1) microglial activation occurred in most studied brain regions, including the hippocampus (HC), thalamus (TM), and hypothalamus (HT); (2) within these three brain regions, the NA-synthesizing enzyme DBH was densely stained in the neuronal fibers; (3) β1-AR and β2-AR, but not β3-AR, are detected in the whole brain, and β1-AR and β2-AR are co-localized with microglial cells, as observed by laser scanning microscopy; (4) β-blocker treatment inhibited microglial activation in terms of morphology and count through the whole brain; α-blockers did not show such effect; (5) unlike WT mice, DKO mice exhibited substantial inhibition of stress-induced microglial activation in the brain.

**Conclusions:**

We demonstrate that neurons/microglia may interact with NA via β1-AR and β2-AR.

## Background

Stress, both physical and psychological, is a risk factor for neurodegenerative disorders such as Parkinson’s disease (PD) and Alzheimer’s disease (AD) [20, 39, 50], which have been associated with neuroinflammation and gliosis. Microglia are immunocompetent brain cells that contribute to neuroinflammation and gliosis. They usually display a ramified morphology, with long dendrites and small cell somas, and are activated by various stimuli, including axotomy, inflammation, and neuronal damage [21, 34]. The microglia can be morphologically classified into several types: ramified, hyper-ramified, reactive, and phagocytic. The phagocytic cell can further be differentiated into transitional (T-stage), motile (M-stage), and locomotor (L-stage) [53, 68]. Once activated, microglia either phagocytize damaged neurons in a cell-to-cell contact fashion [19, 45, 54] or further harm injured neurons by releasing cytotoxic factors, such as nitric oxide, cytokines, chemokines, and reactive oxygen species [10, 18, 36]. Thus, controlling the microglial status is critical for maintaining normal brain activity.

One intriguing feature of microglia is their rapid response to stressful events such as acute [57, 76] or repeated [42, 46, 59, 66] stress, social defeat [35, 52, 72], occlusal teeth disharmony [33], myocardial infarction [48], alcohol [69], sleep deprivation [4, 25], and hypertension [7]. Interestingly, microglial activation is also detected in psychiatric disorders such as depression [65, 75], bipolar disorder [23], and autism [63]. Using an in vivo rodent model, we demonstrated previously that, upon exposure to acute restraint/water immersion stress, microglia immediately become activated [57]. To our knowledge, this report might be the first one demonstrating stress-induced microglial activation [49, 68], although microglial activation has been reported in various models of acute, subacute, and chronic stress [11, 68, 69]. Regardless of the stress type, it is conceivable that microglia respond to “common” signals generated by stress responses. However, the molecular mechanisms underlying stress-related microglial activation remain unknown.

In our previous study, we demonstrated that stress-induced microglial activation occurs within 30 min of exposure to restraint/water immersion stress [57]. This indicates the involvement of fast signals, such as those conveyed by neurotransmitters.

NA is the best documented neurotransmitter in stress experiments. For instance, NA has been reported to increase in the brain in response to various types of stresses including immobilization, foot shock, tail pinch [17, 44, 62]. In addition, administration of β AR agonist, isoproterenol, significantly increased interleukin-1β (IL-1β) in the brain [30, 74], and cultured microglia [64]. Besides, microglial activation induced by repeated social defeat is completely blocked by propranolol, an antagonist of β1 and β2 ARs (β1-AR and β2-AR) [72]. Furthermore, the induction of IL-1β in the hypothalamus (HT) by foot shock stress is blocked by propranolol [8, 9], which also inhibits proinflammatory cytokine production in microglial cells isolated from rats [71]. Collectively, these results suggest that microglia may receive noradrenergic signals in stressed brains. Therefore, we hypothesize that the sympathetic nervous system, most likely noradrenergic neurons, may control the microglial activation status. Here, we demonstrate a possible mechanism for stress-induced microglial activation.

## Methods

### Animals and treatments

**Fig. 1:**
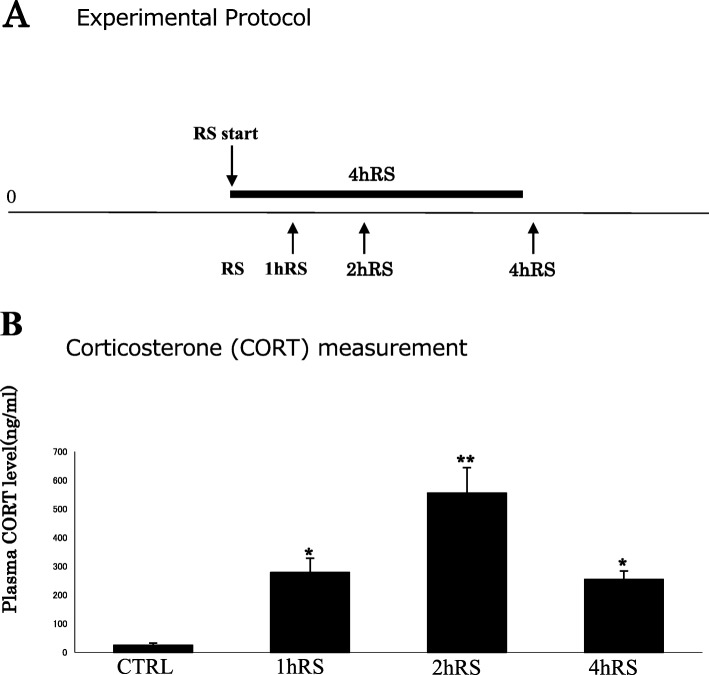
**a** A schematic depiction of the acute RS protocol. Fischer rats were subjected to acute RS for 1–4 h. **b** Plasma corticosterone levels (ng/mL) under control conditions and acute RS. The asterisks indicate the statistical difference between acute RS rats and control rats (^*^p < 0.05, ^**^p < 0.01, *n* = 4)

All procedures were approved by the Institutional Animal Care and Use Committee of the Nippon Medical School (permission no. 27-052; Tokyo, Japan) and were performed in accordance with the National Institutes of Health *Guide for the Care and Use of Laboratory Animals*, aiming to minimize the number of animals used and their suffering. Fischer rats (F344, males, 250–280 g), known to be a stress-sensitive strain, were purchased from Japan Laboratory Animals, Inc. (Tokyo, Japan). For stress experiments, the animals were restrained with wire nets for 1–4 h. Restraint stress (RS) was started at 10:00 a.m. and ended at 11:00 a.m., 12:00 a.m., and 2:00 p.m. (Fig. 1a). The rats were pretreated with the β-blocker propranolol (10 mg/kg) (P0884-1G; Sigma-Aldrich, St. Louis, MO, USA) and the α-blockers prazosin (0.5 mg/kg) (P7791-50MG; Sigma-Aldrich) and yohimbine (3.0 mg/kg) (Y3125-1G; Sigma-Aldrich), which were administered intraperitoneally, 1 h before the experiments. All animals were sacrificed immediately after the RS. Age-matched, unstressed animals, sacrificed immediately after being removed from the animal room, were used as controls. All animals were housed, with Fischer rats two to three and mice two to five in a cage, in a room with controlled temperature (21 ± 1 °C), on a 12:12 light:dark cycle with lights on at 7:00 a.m, with food and water provided ad libitum access.

### Double-knockout mice

Homozygous null mice (Adrb1^tm1Bkk^ Adrb2^tm1Bkk^/J; stock no. 003810) for the Adrb1 and Adrb2 mice were obtained from the Jackson Laboratory and genotypes were confirmed as described by the Jackson Laboratory. Briefly, two separate PCR reactions were set up for each genomic DNA sample, one for adrb1 and one for Adrb2. Both PCR reactions were performed at 95 °C for 2 min, (95 °C for 30 s, 56 °C for 30 s, 72 °C for 45 s), 35 cycles, 72 °C for 5 min, hold at 4 °C. Primers for Adrb1 include WT forward (5′-CTG ATC TGG TCA TGG GAT TG-3′), WT reverse (5′-GCG ATG ACA CAC AGG GTC T-3′), Mutant forward (5′-CGC TGT CCA CAG TGG TTG T-3′) Mutant reverse (5′-TGG CTA CCC GTG ATA TTG CT-3′), Primers for Adrb2 include common reverse (5′-CCG GGA ATA GAC AAA GAC CA-3′), WT forward (5′-ACC AAG AAT AAG GCC CGA GT-3′), and Mutant forward 5′-CAC GAG ACT AGT GAG ACG TG-3′).

### Immunohistochemistry (IHC)

Immunohistochemical analyses were performed as previously described, with minor modifications [51]. The following primary antibodies and dilutions were used: OX-42 (1:1,000; Serotec, Oxford, UK), Iba1 (1:1,000; Wako Pure Chemical Industries, Osaka, Japan), CD11b (1:1,000; Serotec), DBH (1:1,000; Millipore, Bedford, MA, USA), Ki 67 (1:1,000: Abcam), Cleaved caspase 3 (1:1,000: Cell Signaling, MA, USA), β1-adrenergic receptor (AR) (1:1,000; Santa Cruz Biotechnology, Santa Cruz, CA, USA), β2-AR (1:1,000; Santa Cruz Biotechnology), and β3-AR (1:1,000; Santa Cruz Biotechnology). After PBSTx rinses, the sections were incubated with biotinylated secondary antibody (Vector Laboratories, Burlingame, CA, USA) for 60 min at 1:200 in 0.1 M PBST containing 1% BSA. Following rinsing, the sections were exposed to avidin–biotin horseradish peroxidase complex in 0.1 M PBST. Antigens were visualized through reaction with 0.05% 3,3′-diaminobenzidine tetrahydrochloride as a chromogen in PBS and 0.003% hydrogen peroxide for 5 min. The sections were mounted on gelatin-coated slides, dehydrated, and coverslipped with Multi-Mount (Matsunami Glass Ind., Ltd., Osaka, Japan).

For immunofluorescence, the following primary antibodies and dilutions were used: OX-42 (1:200), DBH (1:200), β1-AR (1:200), β2-AR (1:200), and β3-AR (1:200).

### In situ hybridization (ISH)

ISH was performed as previously described, with minor modifications [56, 59]. Tissues were prehybridized in a hybridization solution (50% formamide, 5% dextran sulfate, 1× Denhardt’s solution, 0.25% SDS, 200 μg/mL *E. coli* transfer RNA, 600 mM NaCl, and 1 mM ethylenediaminetetraacetic acid [EDTA]). Digoxigenin- (DIG-) labeled sense and antisense RNA probes were synthesized from the template cDNA (tyrosine hydroxylase (TH), β1-AR, β2-AR, and β3-AR), which was subcloned into pGEM-T Easy Vector (Promega, Madison, WI, USA) using a DIG RNA labeling kit (Roche Diagnostics, Mannheim, Germany). SP6 RNA polymerase was used for antisense probe labeling with SphI (restriction enzyme) cut plasmid, whereas T7 RNA polymerase was used for sense probe labeling with SpeI (restriction enzyme) cut plasmid.

### Reverse transcriptase-polymerase chain reaction (RT-PCR)

Total RNA was isolated from the brain tissues using the RNA extraction buffer ISOGEN (Nippon Gene, Tokyo, Japan). cDNA was synthesized from 2 μg of total RNA using SuperScript III First-Strand Synthesis System (Invitrogen, Carlsbad, CA, USA) according to the manufacturer’s instructions. PCR amplification was performed with Taq PCR polymerase (ABgene, Tokyo, Japan), and the amplified products were dissolved by agarose gel electrophoresis. The sequence of primers used for PCR is as follows: rat GAPDH: 5′-CCT TCA TTG ACC TCA ACT ACA TGG T-3′ and 5′-TCA TTG TCA TAC CAG GAA ATG AGC-3′; rat β1-AR: 5′-CAT CGT GGT GGG TAA CGT GCG G-3′ and 5′-AAA TCG CAG CAC TTG GGG TC-3′; rat β2-AR: 5′-ACC TCC TTT TTG CCT ATC CA-3′ and 5′-TAG GTT TTC GAA GAA GAC CT-3′; rat β3-AR: 5′-TCC CCT CCT TGT GAT GCT-3′ and 5′-AAC GGA CGC GCA CCT TCA-3′. PCR assays comprised an initial 10 min, 94 °C step to activate Taq polymerase, followed by 36 cycles of denaturation at 94 °C for 10 s, annealing at 55 °C for 10 s, and extension at 72 °C for 25 s.

### Quantification of immunoreactivity and ISH

In order to quantify immunohistochemical signals and ISH, the mean optical densities (ODs), defined as the average of the ODs within the target area, were measured from each section using the image analysis software WinROOF (Mitani Corporation, Tokyo, Japan) [51].

### Cell counting, cell size measurement, and Sholl analysis

The Iba1-immunoreactive (Iba1-ir) microglial cells of the hippocampus (HC) (comprising the unilateral DG area), the thalamus (TM), and the HT were counted in a 200 × 200 μm square using WinROOF. For the cell size measurement, all pixels with gray level values below the threshold value were treated as belonging to cell image, and other pixels were treated as background. The appropriate threshold value was determined as the level at which the binary overlay completely covered the entire cell body and processes. Cell surface area was measured using binary images of cells, microglia and astrocyte, with image analysis software (WinROOF). To further evaluate the morphological changes, we performed Sholl analysis for microglial cells as well as astrocytes [13]. Briefly, circles of diameter ranging from 0 to 40 μm, with 10 μm interval, were placed on the imaged cells, with each ring centered on the soma of each single cell. Intersections between the ring and the cell dendrites were counted using the image analysis software LuminaVision (Mitani Corporation, Tokyo, Japan), and the intersection count were averaged for all cells in images from each animal on different experimental conditions [67] (Fig. 6C).

### Enzyme-linked immunosorbent assay (ELISA) for corticosterone measurement

Blood was collected into a tube containing EDTA. After 30 min of centrifugation, the plasma samples were stored at − 80 °C until used for the measurements. Corticosterone levels were measured using an ELISA kit (Cayman Chemical Company, Ann Arbor, MI, USA).

### Statistical analysis

Results are presented as the mean ± standard error of the mean (SEM). All statistical analyses were performed with SPSS software (IBM, Chicago, IL, USA). Statistical significance was determined by Student’s *t* test and one-way and two-way analysis of variance (ANOVA). ANOVA was followed by Bonferroni’s test for multiple comparisons. A level of *p* < 0.05 was considered statistically significant. All results were obtained from four rats and experiments under each condition.

## Results

### Monitoring stress levels with CORT in the plasma of rats exposed to acute stress

First, the stress levels of Fischer rats exposed to acute RS were monitored via the quantification of plasma corticosterone, following the stress procedure (Fig. 1b).

Plasma corticosterone levels significantly increased from 24.0 ± 7.9 ng/mL (under control conditions) to 278.7 ± 49.3 ng/mL at 1 h RS (*n* = 4, *p* < 0.05) and to 554.7 ± 88.8 ng/mL at 2 h RS (*n* = 4, *p* < 0.01), slightly decreasing to 253.9 ± 28.7 ng/mL at 4 h RS (*n* = 4, *p* < 0.05) (Fig. 1b). Consistent with previous reports [28, 29], corticosterone levels peaked in the middle of stress duration. The four groups differed significantly in terms of the stress effect (*n* = 4, *F*(3,12) = 22.479, *p* < 0.001; one-way ANOVA), suggesting that 1–4 h exposure to acute RS effectively induces stress responses in Fischer rats.

### Microglial activation following acute RS

**Fig. 2:**
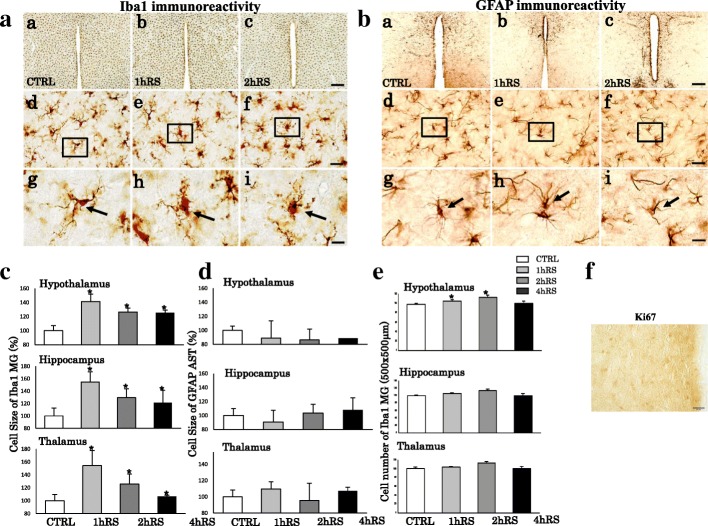
**A**, **B** Morphological changes of Iba1-ir (**A**) and GFAP-ir (**B**) cells following RS in the HT. The upper panels show low-power photomicrographs of Iba1 and GFAP immunoreactivity in the HT of the control (CTRL) (a), 1 h RS (b), and 2 h RS (c). Panels (d), (e), and (f) are enlargements of panels (a), (b), and (c), respectively. Panels (g), (h), and (i) are enlargements of boxed areas (d), (e), and (f), respectively. Arrows indicate resting (g) and activated (h, i) microglia. Scale bar: 200 μm at low-power, 20 μm at middle-power, and 10 μm at high-power magnification. **C**, **D** Histograms showing the quantification of the cell size of Iba1-ir microglia and GFAP-ir astrocytes in the HT, HC, and TM at CTRL, 1 h RS, 2 h RS, and 4 h RS, respectively. **E** Histograms showing the number of Iba1-ir microglia in the HT, HC, and TM at CTRL, 1 h RS, 2 h RS, and 4 h RS, respectively. The asterisks indicate a statistical difference between post-RS and control for each group. ^*^p < 0.05, *n* = 4. Data are presented as means ± SEM. **F** No immunoreactivity to Ki67 was detected in the HT at 1h RS. Scale bar: 10 μm

Next, we investigated microglial morphological changes. Under control conditions, resting microglia are distributed throughout the HT, HC, and TM. Following RS, microglia altered their morphology, becoming enlarged with shorter processes and enlarged soma in the HT, HC, and TM (Fig. 2A). The analysis based on cell size measurement showed a significant increase of Iba1-ir microglial cell size in the HT (*n* = 4, *F*(3,12) = 17.355, *p* < 0.001; one-way ANOVA), HC (*n* = 4, *F*(3,12) = 3.962, *p* < 0.05; one-way ANOVA), and TM (*n* = 4, *F*(3,12) = 3.561, *p* < 0.05; one-way ANOVA) (Fig. 2C). This morphological activation was not observed in astrocytes (Fig. 2B, D). This indicates the occurrence of morphological activation of microglial cells, but not astrocytes, following acute RS in Fischer rats. In addition, RS significantly increased the number of Iba1-ir microglia in the TM (*n* = 4, *F*(3,12) = 4.079, *p* < 0.05; one-way ANOVA), but not in the HT (*n* = 4, *F*(3,12) = 3.066, *p* = 0.069; one-way ANOVA) or HC (*n* = 4, *F*(3,12) = 3.066, *p* = 0.069; one-way ANOVA) (Fig. 2E). With regard to the microglial increases, we found no immunoreactivities to Ki67, a marker for proliferation through mitosis, in the HT that showed significant increase of microglia (Fig. 2F). These results suggest that microglia respond to acute RS by changing the cell morphology and increasing Iba1 expression, presumably not through cell proliferation.

### DBH-ir neuronal fibers in the HT, TM, and HC

Microglia possess ARs [41, 61], suggesting communication with noradrenergic neurons. Therefore, we investigated the immunoreactivity of DBH, the specific enzyme that yields NA from dopamine, in the locus coeruleus (LC), HT, TM, and HC.

**Fig. 3:**
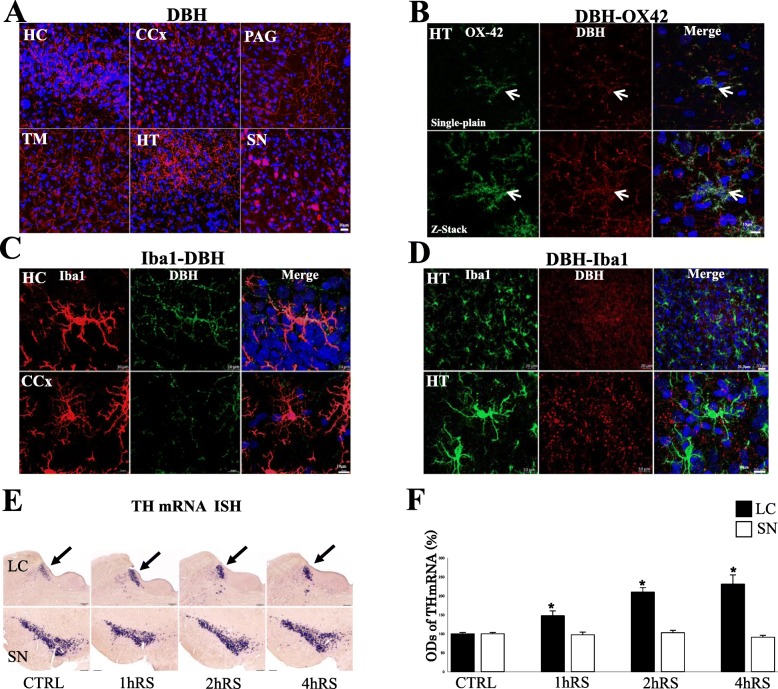
**a** DBH immunofluorescence staining in the HC, TM, CCx, HT, CAG and SN. Scale bar: 10 μm. **b** Double-immunofluorescence staining with DBH (red) and OX-42 (green) in the HT. Upper panels show single-plain images, lower panels showing Z-stack analysis with DBH-ir fibers drawing OX-42-ir microglia as indicated by arrows. Scale bar: 10 μm. **c** Double-immunofluorescence staining with DBH (green) and Iba1 (red) in the HC and CCx. Scale bar: 10 μm. **d** Double-immunofluorescence staining with Iba1 (green) and DBH (red) in the HT, with upper panels at low-power and lower panels at high-power, respectively. Scale bar: 20 μm (upper panels) and 10 μm (lower panels). **e** Th mRNA in situ hybridization in the LC and SN following acute RS as compared to control conditions. Scale bar: 200 μm. **f** Histogram demonstrating the optic densities (O.D.s) of Th mRNA in the LC (black) and SN (white) at each time point (*p < 0.05, **p < 0.01, *n* = 4)

IHC revealed that DBH-ir fibers extended to the HT, TM, and HC (Fig. 3a). DBH-ir fibers were also detected in most brain regions, including the cerebral cortex (CCx), periaqueductal gray (PAG), and substantia nigra (SN) (Fig. 3a).

Double IHC, using OX-42 and DBH, revealed that the fibers of DBH-ir neurons were extended, surrounding microglial cells in the HC, TM, and HT (data not shown). Furthermore, confocal immunofluorescence revealed that, in the HC, OX-42-ir microglia were meticulously surrounded by DBH-ir fibers (Fig. 3b, upper panels). Z-stack analysis, which investigates 3D structures, clearly demonstrated DBH-ir fibers framing the OX-42-ir microglial cells (Fig. 3b, lower panels). Furthermore, Z-stack analysis demonstrated the co-localization of DBH-ir fibers and OX-42-ir microglial cells in other regions such as HC and CCx (Fig. 3c). This finding was also confirmed with a different combination with Iba1-DBH staining which demonstrated the DBH-ir fibers surrounding Iba1-ir microglial cells in the HT (Fig. 3d).

### Noradrenergic neuronal activation in the LC, but not in the SN

It is well known that the cell bodies, projecting DBH-ir axons to a variety of brain regions as shown in Fig. 2A, are located in the LC. To demonstrate the noradrenergic neuronal activation, we measured TH mRNA in the LC and SN. TH mRNA significantly increased in the LC following acute stress (*n* = 4, *F*(3,12) = 21.236, *p* < 0.001; one-way ANOVA) (Fig. 3e, f). In contrast to the LC, no significant change of TH mRNA levels was observed in the SN following acute stress (*n* = 4, *F*(3,12) = 1.126, *p* = 0.377; one-way ANOVA) (Fig. 3e, f). These results demonstrated the activation of noradrenergic neurons in the LC following the acute stress in Fischer rats.

### ARs in the brain

Although both β-ARs (β1-AR, β2-AR, and β3-AR) and α-ARs (α1-AR and α2-AR) are expressed in cultured microglia, the former has been well demonstrated to induce c-AMP elevation [41, 47, 61]. We therefore investigated the expression and distribution of β-ARs in the brain.

**Fig. 4:**
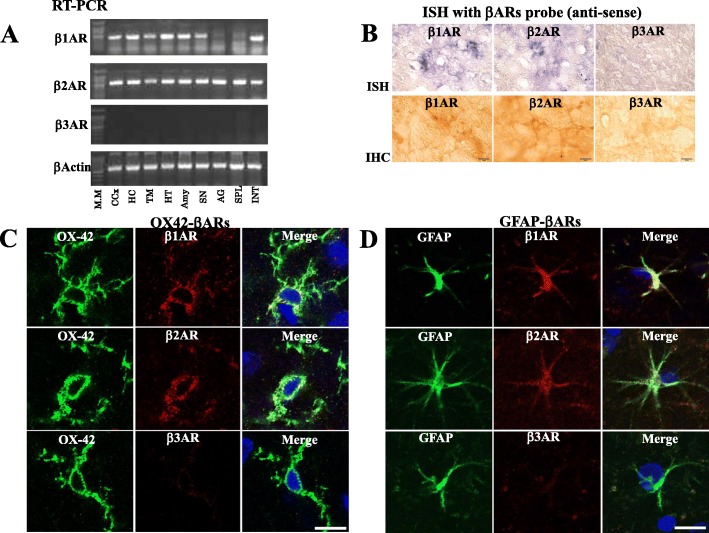
**a** RT-PCR analysis for β1-AR, β2-AR, and β3-AR in the CCx, HC, TM, HT, amygdala (Amy), SN, and peripheral organs, such as the adrenal gland (AG), spleen (SPL), and intestine (INT). β-Actin was used as an internal control. **b** ISH and IHC for β1-AR, β2-AR, and β3-AR in the HT. Scale bar: 10 μm. **c**, **d** Double immunofluorescence for OX-42 and β1-AR, β2-AR, and β3-AR in the HT (**c**) and GFAP and β1-AR, β2-AR, and β3-AR in the HT (**d**), respectively. Scale bar: 20 μm

RT-PCR analysis showed a clear band of β1-AR and β2-AR in each brain region, including the HC, TM, and HT. However, β3-AR was not detected in any brain region (Fig. 4a). ISH showed the expression of β1-AR and β2-AR, but not β3-AR, in the HT (Fig. 4b), which was further confirmed by IHC, showing the existence of β1-AR and β2-AR in glia-like cells (Fig. 4b).

Laser scanning microscopy showed that no immunoreactive β3-AR cells were co-localized with microglia (Fig. 4c) or astrocytes (Fig. 4d). In contrast, β1-AR and β2-AR were co-localized with microglia (Fig. 4c) and astrocytes (Fig. 4d).

### β-Blockers significantly suppress Iba1-ir microglia

**Fig. 5:**
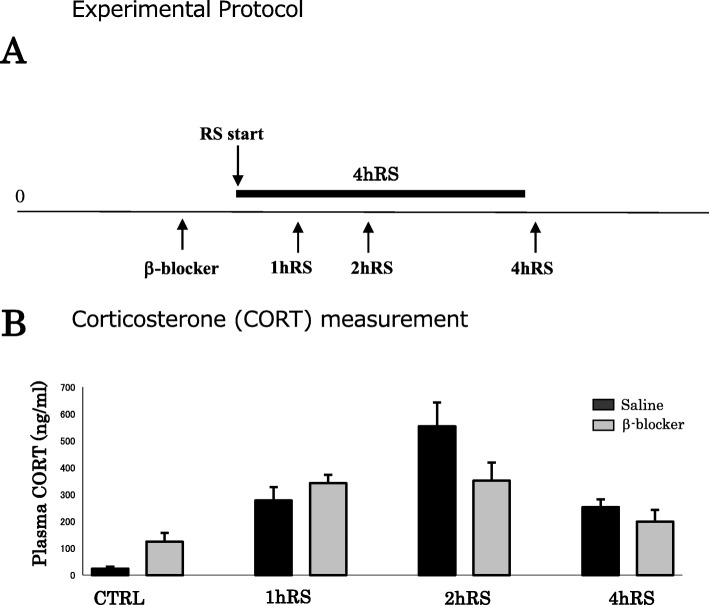
**a** A schematic depiction of the β-blocker treatment protocol. **b** Plasma corticosterone levels (ng/mL) of saline- and β-blocker-treated rats in acute RS. Results are presented as means ± SEM

In order to study the functional involvement of β-ARs, the β-blocker propranolol (10 mg/kg) was administered intraperitoneally 1 h before each stress procedure (Fig. 5a) [72]. Corticosterone levels significantly increased during exposure to stress (*n* = 4, *F*(3,31) = 27.401, *p* < 0.001; two-way ANOVA), and no significant effect of drug treatment was observed (*n* = 4, *F*(1,31) = 0.575, *p* = 0.456; two-way ANOVA) (Fig. 5b).

**Fig. 6:**
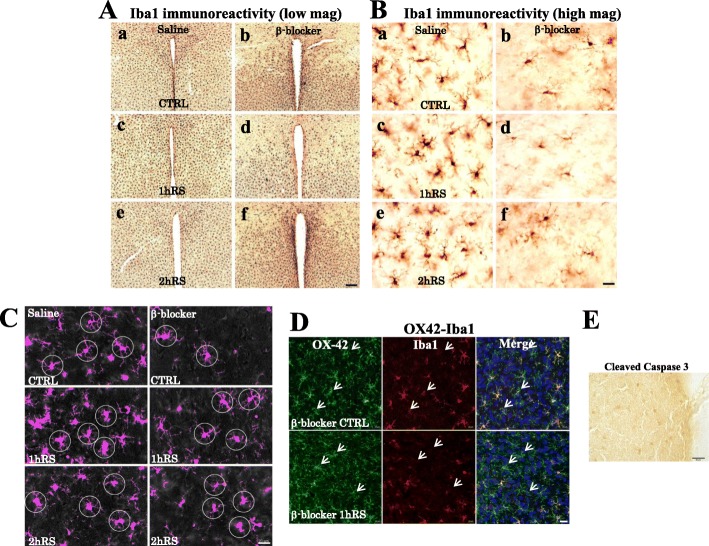
**A** Iba1 IHC for CTRL (a, b), 1 h RS (c, d), and 2 h RS (e, f) after saline and β-blocker treatment, respectively. **B** High-power photomicrographs of Iba1 IHC for CTRL (a, b), 1 h RS (c, d), and 2 h RS (e, f) after saline and β-blocker treatment, respectively. Scale bar: 200 μm in low-power and 20 μm in high-power photos. **C** Circles, 20 μm from the soma, used for Scholl analysis are shown on Iba1-ir microglia at CTRL, 1 h RS, and 2 h RS after saline and β-blocker treatment, respectively. Scale bar: 20 μm. **D** Double-immunofluorescence staining with OX-42 (green) and Iba1 (red) in the HT, with upper panes at CTRL (β-blocker), and 1h RS (β-blocker), respectively. Scale bar: 20 μm. **E** No immunoreactivity to cleaved caspase-3 was found in the HT at 1h RS after propranolol treatment. Scale Bar: 20 μm

**Fig. 7:**
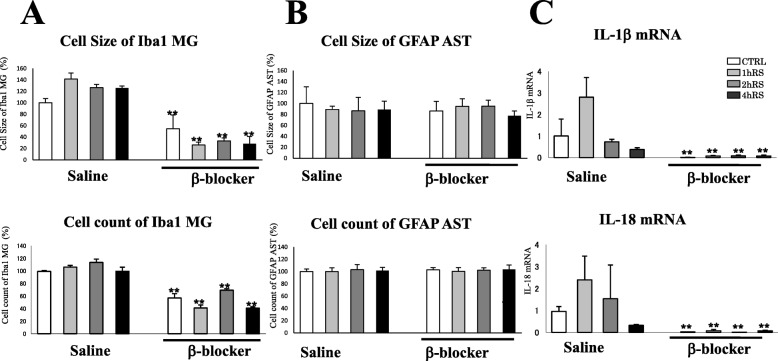
**a**, **b** Histograms demonstrating the cell size (upper) and cell count (lower) of Iba1-ir microglia (**a**) and GFAP-ir astrocytes (**b**) in the HT at CTRL, 1 h RS, 2 h RS, and 4 h RS, respectively, as compared to β-blocker treatment. **c** Real-time PCR analysis for IL-1β and IL-18 mRNAs. GAPDH mRNA was used as an internal control. An arbitrary value of 1 was assigned to the levels of IL-1β/GAPDH and IL-18/GAPDH under control conditions. The asterisks indicate a statistical difference between saline-treated and β-blocker-treated rats at each time point (^*^p < 0.05, ^**^p < 0.01, *n* = 4)

Furthermore, we evaluated cells morphology by employing parameters such as cell size, cell count, and intersections. In contrast to saline-treated rats, the microglial density looked sparse in the HT of propranolol-treated rats (Fig. 6A, B). The morphological activation of microglia differed significantly between saline- and propranolol-treated rats (*n* = 4, *F*(1,31) = 193.082, *p* < 0.001; two-way ANOVA). Propranolol significantly suppressed the microglial density at CTRL (*n* = 4, *p* < 0.01), 1 h RS (*n* = 4, *p* < 0.01), 2 h RS (*n* = 4, *p* < 0.01), and 4 h RS (*n* = 4, *p* < 0.01), compared with saline-treated rats (Fig. 7a). In addition, there was a significant effect of propranolol on the number of microglia (*n* = 4, *F*(1,31) = 374.025, *p* = 0.001; two-way ANOVA) in that the HT of propranolol-treated rats had a significantly lower microglial count at CTRL (*n* = 4, *p* < 0.01), 1 h RS (*n* = 4, *p* < 0.01), 2 h RS (*n* = 4, *p* < 0.01), and 4 h RS (*n* = 4, *p* < 0.01) than that of saline-treated rats (Fig. 7a). There was no significant effect of propranolol treatment on astrocyte cell size (*n* = 4, *F*(1,31) = 0.060, *p* = 0.809; two-way ANOVA) or cell count (*n* = 4, *F*(1,31) = 0.053, *p* = 0.819; two-way ANOVA) (Fig. 7b)

**Fig. 8:**
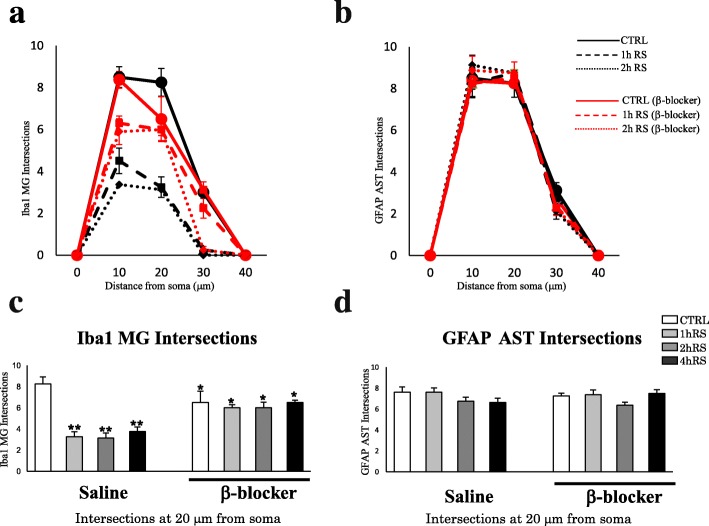
**a**, **b** Scholl analysis of microglia and astrocytes in the HT at CTRL, 1 h RS, and 2 h RS, respectively, as compared to β-blocker treatment. **c**, **d** Histograms demonstrating the intersections count of Iba1-ir microglia (**c**) and GFAP-ir astrocytes (**d**) at the distance of 20 μm from soma. The asterisks indicate a statistical difference between saline-treated and β-blocker-treated rats at each time point (^*^p < 0.05, ^**^p < 0.01, *n* = 4)

Moreover, we analyzed morphological changes with Sholl analysis. As shown in Fig. 8a, there was some difference on the count of intersections between 10 and 20 μm on the distance from soma. For instance, at 10 μm from soma, there was no difference on the intersection numbers in the control conditions between saline and β-blocker treatment. In the stressed conditions, such as 1 h RS or 2 h RS, there was decrease on the intersection numbers both in saline and β-blocker-treated rats. At 20 μm from soma, there were significant differences both in control and stressed conditions between saline and β-blocker treatment. In particular, in saline-treated rats, there was a significant decrease of the count of intersections on microglial cells in stressed conditions (*n* = 4, *F*(3,12) = 18.000, *p* < 0.001; one-way ANOVA). However, in propranolol-treated rats, there was no significant change between control and stressed conditions in propranolol-treated rats based on Sholl analysis (*n* = 4, *F*(3,12) = 0.425, *p* = 0.739; one-way ANOVA) (Fig. 8a, c). In fact, there was already a significant decrease of intersection counts even in control conditions (Fig. 8c). The decrease of intersections of microglia in control conditions in propranolol treatment is likely to be due to cellular shrinkage as shown in Fig. 6B, not to the hypertrophic changes.

With regard to astrocytes, there was no significant effect of stress (*n* = 4, *F*(3,12) = 0.284, *p* = 0.836; one-way ANOVA) or propranolol (*n* = 4, *F*(3,12) = 0.393, *p* = 0.760; one-way ANOVA) on the intersections of GFAP-ir astrocytes (Fig. 8b, d). There were also no significant effects of stress or propranolol in the HC, TM, and SN (data not shown).

Curiously, during the course of the experiment we found that there are two types of microglia: OX-42^+^-Iba1^+^ and OX-42^+^-Iba1^−^ (Fig. 6D). As to the cause behind the drastic decrease of Iba1-ir microglial cells following propranolol, we found no evidence of cleaved caspase-3 staining (6E), suggesting that the change may be not through apoptosis.

Next, we quantified the proinflammatory markers IL-1β and IL-18 using real-time PCR and found that propranolol significantly suppressed the expression of IL-1β and IL-18 mRNA at CTRL (*n* = 4, *p* < 0.01), 1 h RS (*n* = 4, *p* < 0.01), 2 h RS (*n* = 4, *p* < 0.05), and 4 h RS (*n* = 4, *p* < 0.05), as compared to saline-treated rats (Fig. 7c).

### α-Blockers significantly increase Iba1-ir microglia

**Fig. 9:**
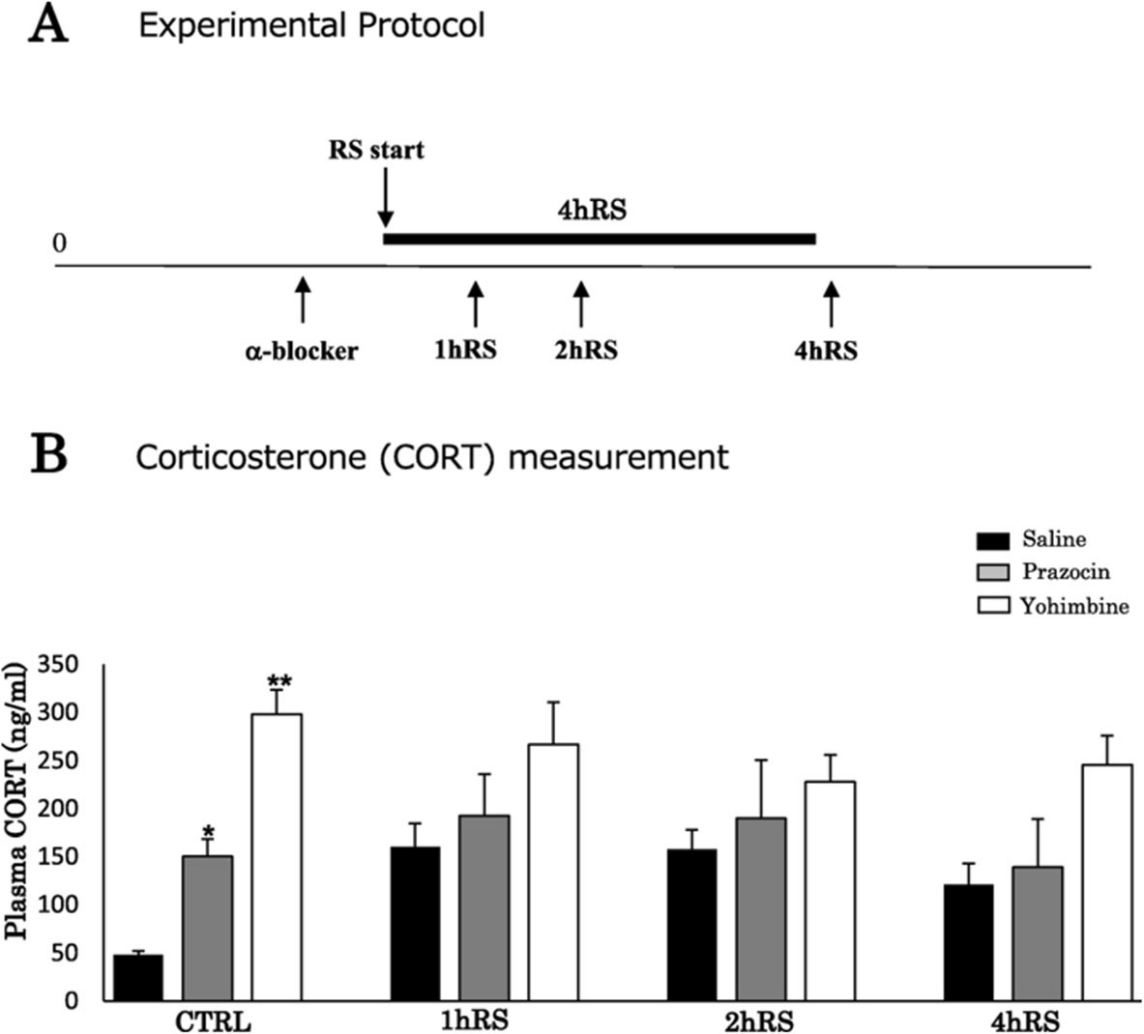
**a** A schematic depiction of the α-blocker treatment protocol with prazosin and yohimbine. **b** Plasma corticosterone levels (ng/mL) of saline- and α-blocker-treated rats in acute RS. The asterisks indicate a statistical difference between saline- and yohimbine-treated rats following acute RS at each time point (^*^p < 0.05, ^**^p < 0.01, *n* = 4). Results are presented as means ± SEM

In order to study the functional involvement of α-ARs, the α1-AR blocker prazosin (0.5 mg/kg) [32] and the α2-AR blocker yohimbine (3.0 mg/kg) [61] were administered intraperitoneally 1 h before each stress procedure (Fig. 9a).

The levels of corticosterone after saline, prazosin, and yohimbine treatment did not increase significantly with stress exposure (*n* = 4, *F*(3,47) = 1.311, *p* = 0.286; two-way ANOVA) (Fig. 9b). However, a significant difference in the corticosterone levels was found among saline- and α-blocker-treated rats under control conditions (*n* = 4, *F*(2,47) = 22.303, *p* < 0.001; two-way ANOVA) (Fig. 9b).

**Fig. 10:**
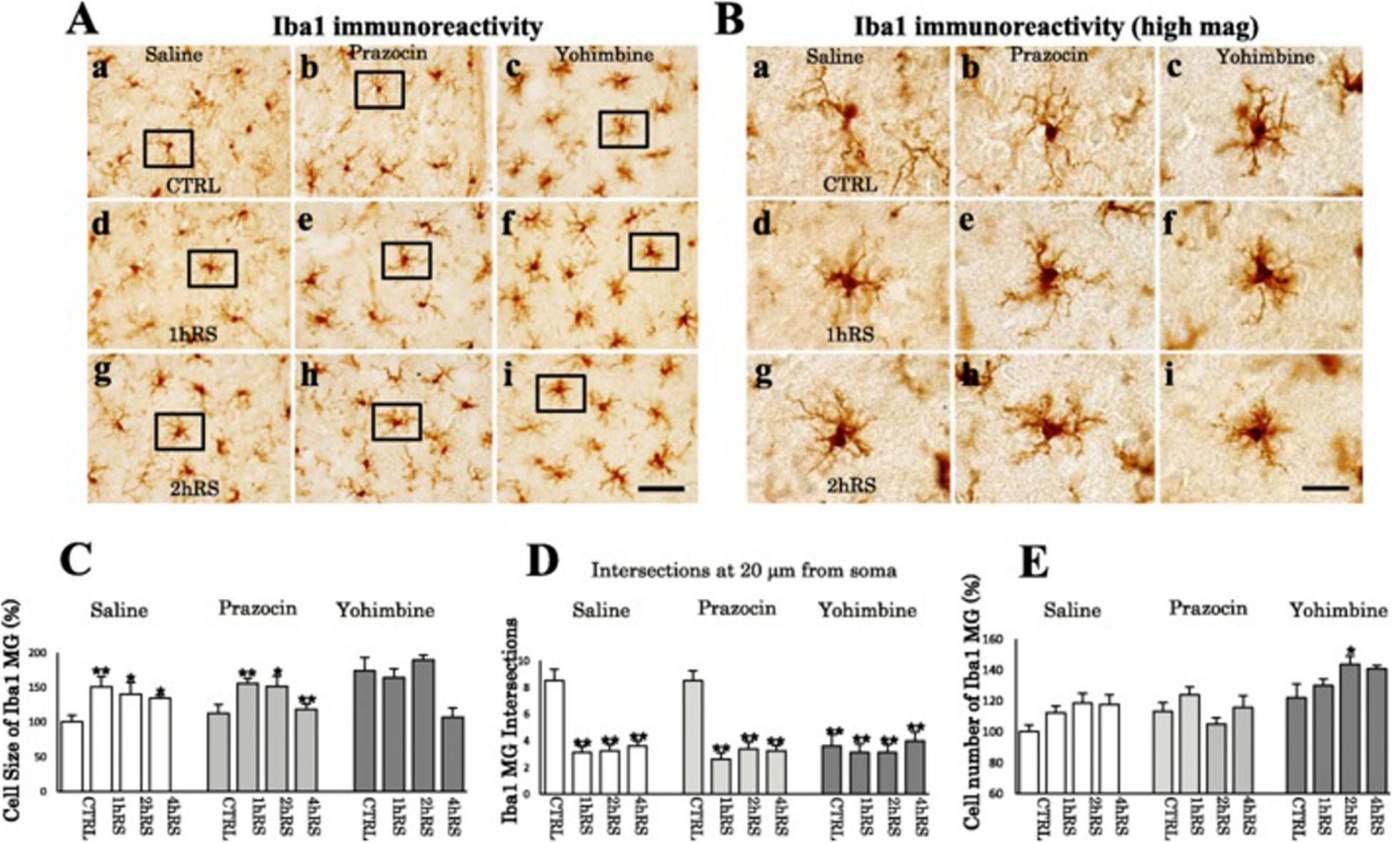
**A** Iba1 IHC for CTRL (a–c), 1 h RS (d–f), and 2 h RS (g–i) after saline and α-blocker treatment, respectively. **B** Enlargement of the boxed areas in **A**. Scale bar: 50 μm in **A** and 20 μm in **B**. **C** Histogram demonstrating the cell size of Iba1-ir microglia in the HT at CTRL, 1 h RS, and 2 h RS following saline and α-blocker treatment. **D** Histogram showing the intersections count. **E** Histogram demonstrating the cell count of Iba1-ir microglial cells in the HT at CTRL, 1 h RS, and 2 h RS following saline and α-blocker treatment. The asterisks indicate a statistical difference between CTRL and stressed rats (^*^p < 0.05, ^**^p < 0.01, *n* = 4)

Microglia were significantly activated in α-blocker-treated rats (*n* = 4, *F*(3,47) = 10.447, *p* < 0.001; two-way ANOVA), and the morphological activation of saline-, prazosin-, and yohimbine-treated rats differed significantly (*n* = 4, *F*(2,47) = 7.638, *p* < 0.001; two-way ANOVA). In prazosin-treated rats, microglial activation occurred in a similar pattern to that in saline-treated rats based on cell size and dendrite intersections (Fig. 10A–D). In contrast, microglial activation was detected even under control conditions, occurring more clearly in yohimbine-treated rats than in prazosin-treated rats, following RS (Fig. 10A–D). In addition, the microglial cell count increased significantly with time (*n* = 4, *F*(3,47) = 4.138, *p* < 0.05; two-way ANOVA), differing significantly among groups (*n* = 4, *F*(2,47) = 24.203, *p* < 0.001; two-way ANOVA) (Fig. 10E). These results suggest that α2-AR blockade with yohimbine facilitated microglial activation.

### Comparison of stress-induced microglial activation between wild-type (WT) and β1-AR and β2-AR double-knockout (DKO) mice

**Fig. 11:**
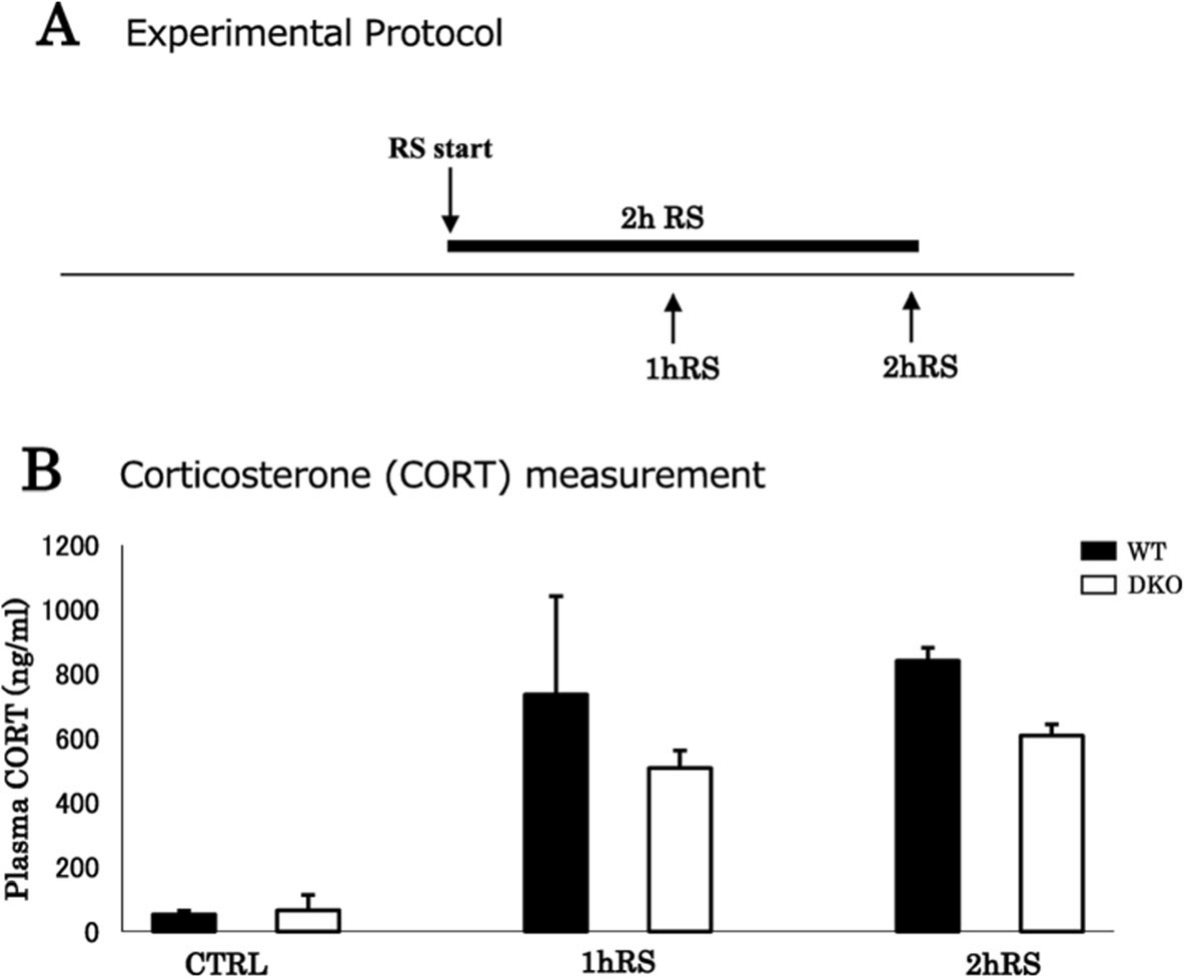
**a** A schematic depiction of the stress protocol for WT and DKO mice. **b** Plasma corticosterone levels (ng/mL) of WT and DKO mice following acute RS (*n* = 4). Data are presented as means ± SEM

Lastly, we compared acute stress-induced microglial activation between WT and DKO mice lacking β1-AR and β2-AR (Fig. 11a).

Corticosterone levels increased significantly upon exposure to acute RS (*n* = 4, *F*(2,23) = 20.382, *p* < 0.001; two-way ANOVA), with no significant difference between WT and DKO mice (*n* = 4, *F*(1,23) = 2.670, *p* = 0.120; two-way ANOVA) (Fig. 11b).

**Fig. 12:**
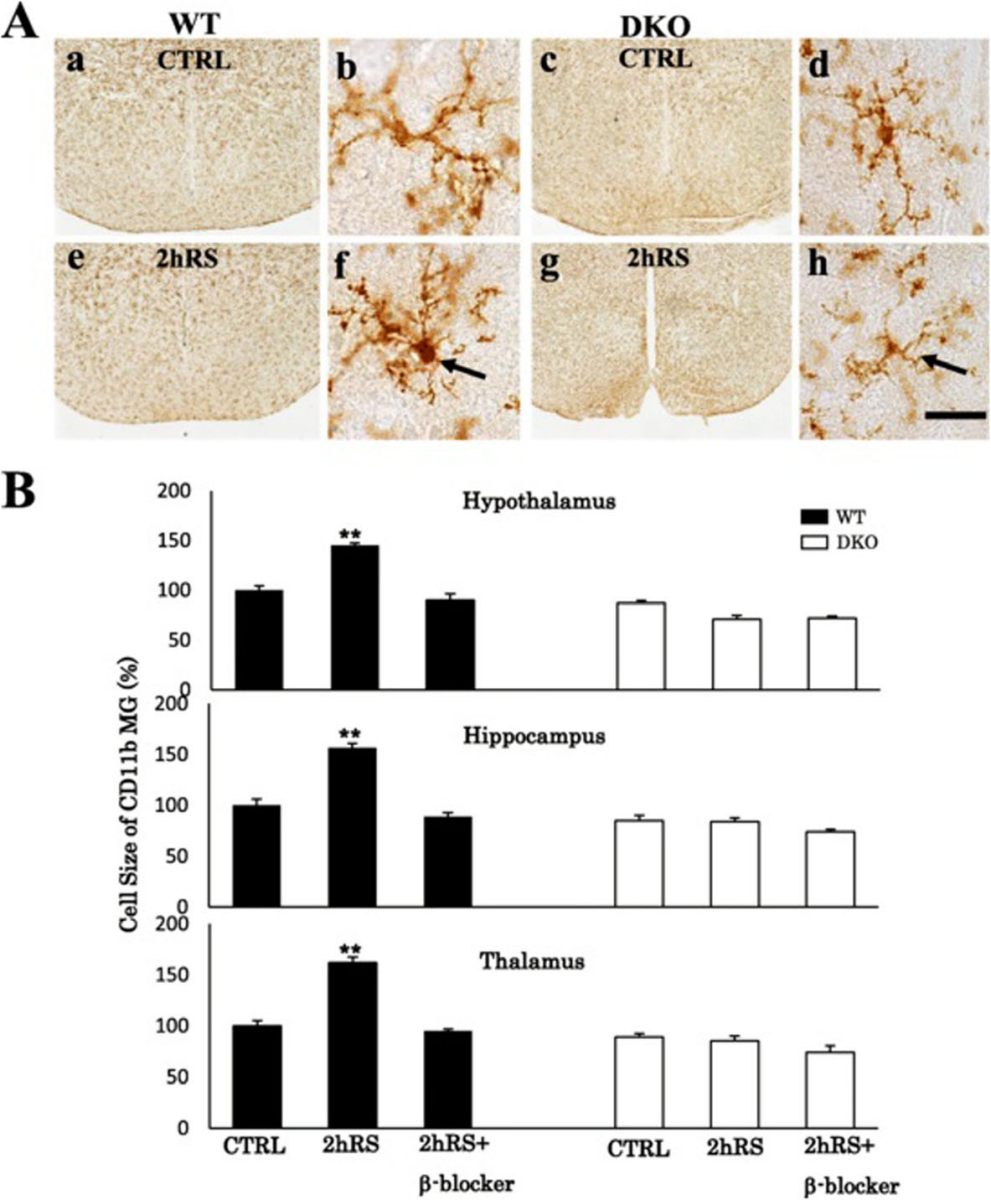
**A** CD11b IHC for CTRL in WT (**a**) and DKO (**c**) mice and for 2 h RS in WT (**e**) and DKO (**f**) mice in low-power photographs and for CTRL in WT (**b**) and DKO (**d**) mice and for 2 h RS in WT (**f**) and DKO (**h**) mice in high-power photographs. Scale bar: 500 μm in low-power and 20 μm in high-power photos. **B** Histograms demonstrating the cell size of CD11b-immunoreactive microglia of WT and DKO mice in the HT, HC, and TM following acute 2 h RS and 2 h RS with β-blocker treatment. The asterisks indicate the statistical difference between acute RS rats and control rats (^**^p < 0.01, *n* = 4)

Under control conditions, the microglia maintained a resting morphology with small cell somas and thin processes in both WT and DKO mice (Fig. 12A). In WT mice, a single 2-h session transformed resting microglia into activated microglia, presenting enlarged cell somas and thick processes, in the HT (*n* = 4, *p* < 0.01), HC (*n* = 4, *p* < 0.01), and TM (*n* = 4, *p* < 0.01) (Fig. 12B). In contrast, in DKO mice, a single 2-h session failed to show microglial activation in the HT, TM, and HC (Fig. 12B). Two-way ANOVA based on cell size revealed that the stress-induced microglial activation was significantly inhibited in the DKO mice in the HT (*n* = 4, *F*(1,15) = 221.618, *p* < 0.001), TM (*n* = 4, *F*(1,15) = 108.529, *p* < 0.001), and HC (*n* = 4, *F*(1,15) = 108.315, *p* < 0.001) (Fig. 12B).

In WT mice, β-blocker treatment significantly inhibited the morphological microglial activation in the HT (*n* = 4, *p* < 0.01), TM (*n* = 4, *p* < 0.01), and HC (*n* = 4, *p* < 0.01) (Fig. 12B).

## Discussion

The main finding of the present study was that microglial activation, as represented by enlarged Iba1-ir cell surface areas in the HT, HC, and TM, was significantly inhibited by pretreatment with the β-blocker propranolol. This finding was further confirmed by impaired, stress-induced, microglial activation in DKO mice, which contrasted sharply with that of WT mice.

Regarding the mechanism of neuronal–glial interaction, we demonstrated that fibers immunoreactive to DBH extend into wide brain regions, including the HC, TM, and HT. This result is consistent with those of previous studies which demonstrated extensive branched axons providing the main source of NA throughout the brain including neocortex, amygdala, cerebellum and spinal cord [37, 40]. In addition to a variety of neuropeptides including neuropeptide Y, somatostatin, cholecystokinin, and galanin, the LC contains two enzymes, DBH and TH, which are critically involved in NA biosynthesis [5, 51]. Therefore, it is crucial to know how DBH and TH respond to the acute RS. Importantly, we found the density of DBH-ir neurons in the LC increased significantly after 2 h RS (data not shown). In addition, mRNA of TH was shown to significantly increase following the RS only in the LC. Thus, these results suggest the activation of the noradrenergic neurons. On the other hand, it has been reported that microglial cells possess receptors for NA, such as β1-AR, β2-AR, β3-AR, α1-AR, and α2-AR [41, 61, 72]. In the present study, confocal microscopic Z-stack analysis revealed that DBH-ir fibers edged microglial cells. In previous reports based on electron microscopy, it has been reported that noradrenergic synapses release NA into the extracellular fluid, diffusing the neurotransmitter into the surrounding synaptic clefts [2, 3]. Moreover, confocal microscopy showed that both β1-AR and β2-AR, but not β3-AR, were co-localized with OX-42-ir microglia in the brain. Taken together, these results suggest that microglial cells possessing β1-AR and β2-AR may receive adrenergic signals from noradrenergic neurons.

In the present study, we investigated the role of β-ARs using the β-blocker propranolol prior to acute RS and observed that microglial activation triggered by acute RS was substantially inhibited in the HT, HC, and TM. In addition, pretreatment with propranolol significantly decreased the number of Iba1-ir microglial cells in those regions. These findings are consistent with those of previous studies demonstrating that propranolol inhibits microglial activation following various stresses, such as social disruption stress [72] and inescapable foot shock [30]. It was also reported that the stress-associated increase of IL-1β mRNA is inhibited by propranolol in the central nervous system [8]. Therefore, we suggest that stress-induced microglial activation may occur through β-ARs.

On the other hand, and against our expectations, microglia were further activated by pretreatment with the α2-AR blocker yohimbine. This effect was stronger than that of the α1-AR blocker prazosin. In this study, it was also found that the levels of plasma corticosterone were elevated in animals treated with those α-AR blockers, which supports the fact that the hypothalamic pituitary adrenal (HPA) axis may be activated by pretreatment with α-AR blockers. In fact, this finding is consistent with those of previous studies showing upregulated corticosterone levels in prazosin- or yohimbine-treated rats [38, 55]. Importantly, the LC receives afferent neurons from the HT [5], causing NA to increase through the activated HPA axis. In addition, α2-AR blockade in chronic, unexpected, mild stress increases the level of NA in the brain [70]. It is well established that the α2-AR plays a role as a presynaptic inhibitory receptor regulating neurotransmitters’ release [1, 12, 22]. Therefore, it is possible that the enhanced microglial activation by yohimbine treatment may be induced by the increased release of NA from the synaptic terminals. The results obtained with α-blockers, such as prazosin and yohimbine, further suggest that stress-induced microglial activation may be achieved through NA upregulation.

Although the involvement of α-ARs in stress-induced microglial activation cannot be excluded, the effect of propranolol, a β1-AR and β2-AR blocker, was more predominant than those of blockers for α1-AR and α2-AR. Therefore, we used DKO mice lacking β1-AR and β2-AR in order to study the functional involvement of β1-AR and β2-AR in microglial activation. Microglial activation was first confirmed in WT mice exposed to 2 h RS. IHC demonstrated that, in DKO mice, the intensities of CD11b immunoreactivities, as a marker of morphological microglial activation in mice, were significantly suppressed in the HT, HC, and TM following acute RS. The level of corticosterone was relatively decreased in DKO mice, as compared to WT mice. Since corticosterone limits microglial activation [58], it is unlikely that the microglial suppression observed in DKO mice may have derived from the corticosterone levels. In addition, the stress-induced microglial activation observed in WT mice was significantly suppressed by propranolol. Intriguingly, there was no significant change in microglial morphology between WT and DKO mice under control conditions, suggesting that β-ARs may not be involved in regulation of resting microglia. As a whole, the present study demonstrates that deletion of β1-AR and β2-AR genes substantially suppresses acute stress-induced microglial activation in the brain. Thus, β1-AR and β2-AR may directly transmit signals, presumably NA, resulting in stress-induced microglial activation.

In several studies, the anti-inflammatory effects of NA have been reported, which contradicts the results of our study. For instance, in cultured microglia, NA and isoproterenol, a β-AR agonist, inhibit proinflammatory markers, such as IL-1β, IL-6, and iNOS mRNA, following LPS treatment (1.0 μg/mL), through the inhibition of NF-κB translocation [27]. In addition, NA depletion in aged rats’ HC has been found to aggravate inflammation following LPS treatment (0.75 mg/kg) [6], whereas the proinflammatory effects of NA have been demonstrated in several studies. For instance, in cultured microglia, administering isoproterenol significantly increases the induction of IL-1β mRNA [64]. β2-AR stimulation leads to an increase of IL-1β and IL-6 [60]. These anti- and proinflammatory effects of NA comprise an intriguing paradox. One aspect common to the abovementioned studies is that experiments showing an anti-inflammatory effect of NA are mostly conducted in combination with LPS treatment, which induces NF-κB translocation into the nucleus. Unlike those experiments, we did not use LPS in the present study. Considering the existence of an alternative mechanism to the conventional PKA/NF-κB-dependent pathway [47], such differential pathway might contribute to the differential effects of NA. It is conceivable that such alternative pathway may underlie the proinflammatory effects of NA.

Microglia play distinctive roles depending on their morphologies. Under resting conditions, microglia with ramified, long, thin processes play constructive roles, such as debris clearance, neuronal support, and synaptic monitoring and remodeling [14, 15]. In contrast, under morphological activation, microglia become hypertrophic with short and thick processes and play various harmful roles, such as cytokine and superoxide release, neurotoxicity, and phenotypic polarization [10, 18, 36]. It is, therefore, critical to keep microglia quiescent and at a resting condition throughout the brain. Importantly, the β-blocker propranolol has been shown to effectively ameliorate neurodegenerative disorders, such as AD and PD. In particular, propranolol (5 mg/kg) has been shown to reduce cognitive deficits and amyloid and tau pathology in a model of AD [16]. In addition, microglial inhibition during sleep is reported to be critical for preventing cognitive dysfunction, via clearance of metabolic waste [4, 24–26, 43, 73]. Furthermore, β-blockers are reported to prevent anxiety-like behaviors through microglial inhibition [72]. Intriguingly, microglial inhibition by minocycline was found to lead to emotional stability [31]. Thus, controlling the microglial status may contribute to a variety of brain functions, ranging from cognition to mental activity. We therefore suggest that β-blockers, rather than α-blockers, are critical for maintaining the microglial status.

## Conclusions

In the present study, we demonstrated that neurons/microglia may interact with NA throughout the brain via β1-AR and β2-AR. NA may therefore be one of the neurotransmitters regulating microglial activation in the brain. β-blockers may effectively treat neurological disorders associated with microglial activation.

## Data Availability

All data generated or analyzed during this study are included in this work.

## Abbreviations

AD: Alzheimer’s disease
ANOVA: Analysis of variance
AR: Adrenergic receptor
CORT: Corticosterone
CTRL: Control
DBH: Dopamine beta hydroxylase
DG: Dentate gyrus
DKO: Double-knockout mouse
ELISA: Enzyme-linked immunosorbent assay
HC: Hippocampus
HPA: Hypothalamic pituitary adrenal axis
HT: Hypothalamus
IHC: Immunohistochemistry
ISH: In situ hybridization
LC: Locus ceruleus
LPS: Lipopolysaccharide
NA: Noradrenaline
OD: Optic density
PD: Parkinson’s disease
RS: Restraint stress
SN: Substantia nigra
TH: Tyrosine hydroxylase
TM: Thalamus
WT: Wild-type mouse
